# A human stomach cell type transcriptome atlas

**DOI:** 10.1186/s12915-024-01812-5

**Published:** 2024-02-14

**Authors:** S. Öling, E. Struck, M. Noreen-Thorsen, M. Zwahlen, K. von Feilitzen, J. Odeberg, F. Pontén, C. Lindskog, M. Uhlén, P. Dusart, L. M. Butler

**Affiliations:** 1 Department of Clinical Medicine, Translational Vascular Research, The Arctic University of Norway, 9019 Tromsø, Norway; 2 Science for Life Laboratory, Department of Protein Science, Royal Institute of Technology (KTH), 171 21 Stockholm, Sweden; 3 The University Hospital of North Norway (UNN), 9019 Tromsø, Norway; 4 Department of Haematology, Coagulation Unit, Karolinska University Hospital, 171 76 Stockholm, Sweden; 5 Department of Immunology, Genetics and Pathology, Science for Life Laboratory, Uppsala University, 752 37 Uppsala, Sweden; 6 Clinical Chemistry and Blood Coagulation Research, Department of Molecular Medicine and Surgery, Karolinska Institute, 171 76 Stockholm, Sweden; 7 Clinical Chemistry, Karolinska University Laboratory, Karolinska University Hospital, 171 76 Stockholm, Sweden

**Keywords:** Cell profiling, Gene enrichment, Bulk RNAseq, Stomach

## Abstract

**Background:**

The identification of cell type-specific genes and their modification under different conditions is central to our understanding of human health and disease. The stomach, a hollow organ in the upper gastrointestinal tract, provides an acidic environment that contributes to microbial defence and facilitates the activity of secreted digestive enzymes to process food and nutrients into chyme. In contrast to other sections of the gastrointestinal tract, detailed descriptions of cell type gene enrichment profiles in the stomach are absent from the major single-cell sequencing-based atlases.

**Results:**

Here, we use an integrative correlation analysis method to predict human stomach cell type transcriptome signatures using unfractionated stomach RNAseq data from 359 individuals. We profile parietal, chief, gastric mucous, gastric enteroendocrine, mitotic, endothelial, fibroblast, macrophage, neutrophil, T-cell, and plasma cells, identifying over 1600 cell type-enriched genes.

**Conclusions:**

We uncover the cell type expression profile of several non-coding genes strongly associated with the progression of gastric cancer and, using a sex-based subset analysis, uncover a panel of male-only chief cell-enriched genes. This study provides a roadmap to further understand human stomach biology.

**Supplementary Information:**

The online version contains supplementary material available at 10.1186/s12915-024-01812-5.

## Background

The gastrointestinal (GI) tract is a multiple organ system which can be divided into upper and lower parts, the physical properties and cellular characteristics of which reflect their different roles in digestion, absorption of nutrients, and excretion of waste products [1–3]. The stomach, a hollow muscular organ in the upper GI tract, produces an array of acids and gastric enzymes, acting as a reservoir for the mechanical and chemical digestion of ingested food [4]. The constituent cell types of the stomach include parietal cells, chief cells, gastric mucous cells, gastric enteroendocrine cells, mitotic cells, endothelial cells, fibroblasts, and various immune cells [5, 6]. In contrast to lower sections of the GI tract, descriptions of the cellular transcriptional landscape in the stomach are lacking, with this organ absent from large-scale single-cell sequencing (scRNAseq) initiatives, such as Tabula Sapiens [7] and the Human Cell Atlas [8]. Where scRNAseq has been used to profile gene expression in the adult stomach, studies have typically focused on specific cell types, such as the epithelia [9, 10], or in pathological states such as gastric cancer [11–14]. Whilst scRNAseq studies provide high resolution of individual cell (sub)type gene expression profiles, challenges remain, including artefactual modification of gene expression due to cell removal and processing [15–17], compromised read depth, and difficulties with data interpretation [18, 19]. As a limited number of biological replicates are typically analysed, underestimation of biological variance can increase the likelihood of potential false discoveries [20, 21].

Non-coding RNA is emerging as a novel, important class of molecules, involved in the maintenance of healthy stomach tissue and the development and progression of gastric cancer [22, 23], but to date, there is no overall description of stomach cell type-enriched non-coding RNAs.

Here, we analysed 359 bulk RNAseq human stomach samples to identify over 1600 genes with cell type-enriched expression, using our previously developed integrative correlation analysis [24–26]. Gastric mucous cells had the highest number of predicted protein-coding and non-coding enriched genes and represented the primary site of expression of genes that were tissue enriched in the stomach over other tissue types. Gastric enteroendocrine cells expressed a panel of non-coding genes that are also selectively expressed in pancreatic and intestinal endocrine cells, indicating a common function in these cell types. Several of the identified cell type-enriched non-coding genes have previously been associated with the progression of gastric cancer, but until now, the cell type site of expression had not been described. Sex subset analysis revealed a high global similarity in cell type transcriptomes between males and females, but a panel of chief cell-enriched Y-linked genes were identified. Data is available through the Human Protein Atlas (HPA) portal (www.proteinatlas.org/humanproteome/tissue+cell+type/stomach).

## Results

### Identification of cell type transcriptome profiles in stomach

#### Cell type reference transcripts correlate across unfractionated RNAseq data

**Fig. 1:**
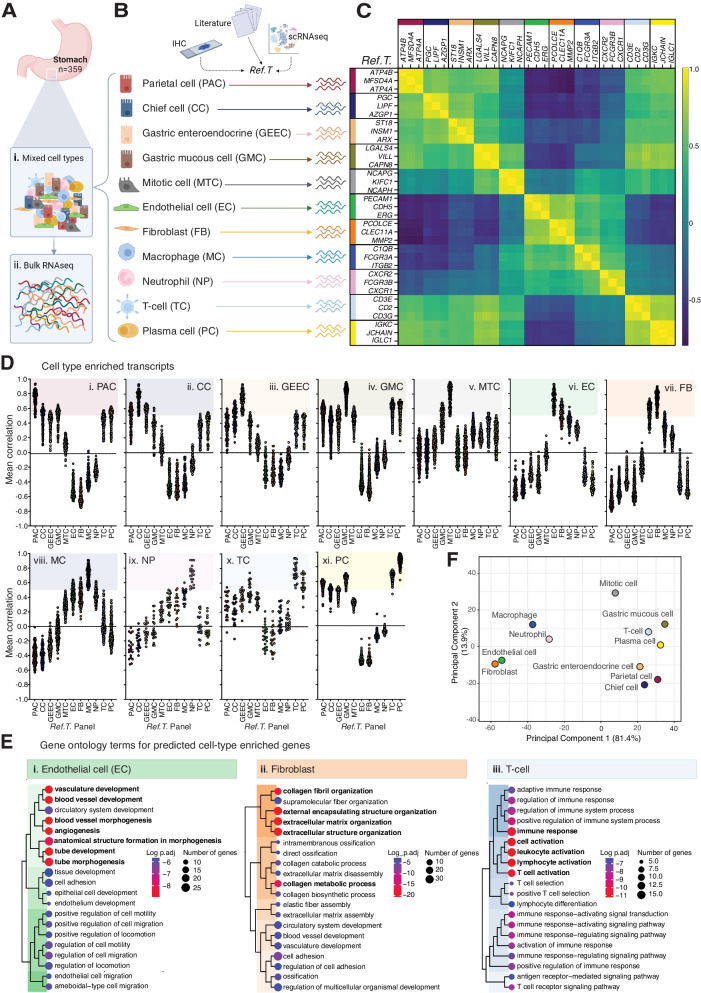
Integrative co-expression analysis can resolve constituent cell type identities from unfractionated human stomach tissue RNAseq data. (**A**) RNAseq data for 359 unfractionated human stomach samples were retrieved from GTEx V8. Each sample contained (i) mixed cell types, which contributed (ii) differing proportions of sequenced mRNA. (**B**) To profile cell type-enriched transcriptomes, constituent cell types were identified and candidate marker genes (´reference transcripts´ [Ref.T.]) for virtual tagging of each were selected, based on in house tissue protein profiling and/or existing literature and datasets. (**C**) Matrix of correlation coefficients between selected Ref.T. across the sample set. (**D**) Mean correlation coefficients of genes above designated thresholds for classification as cell-type enriched in stomach: (i) parietal cells [PC], (ii) chief cells [CC], (iii) gastric enteroendocrine cells [GEEC], (iv) gastric mucous cells [GMC], (v) mitotic cells [MTC], (vi) endothelial cells [EC], (vii) fibroblasts [FB], (viii) macrophages [MC], (ix) neutrophils [NP], (x) T-cells [TC], (xi) plasma cells [PC] with all Ref.T. panels. (**E**) Over-represented gene ontology terms among genes predicted to be: (i) endothelial cell, (ii) fibroblast or (iii) T-cell enriched. (**F**) Principal component analysis of correlation profiles of cell type enriched genes. See also Table S1 Tab 1 and 2 and Figure S1 for method overview

To identify stomach cell type-enriched transcriptome profiles, we conducted an analysis based on our previously developed method [24–26], using human stomach bulk RNAseq data (*N* = 359) from the Genotype-Tissue Expression (GTEx) portal V8 [27] (see Additional file 1: Fig. S1 for the method overview). Each sample was unfractionated and thus contained a mix of cell types (Fig. 1A.i), which contribute differing proportions of transcripts subsequently measured by RNAseq (Fig. 1A.ii) (Additional file 1: Fig. S1A). For each major constituent stomach cell type, candidate cell type-specific genes (termed ‘reference transcripts’ [*Ref.T.*]) were selected based on (i) our in-house proteomic profiling of stomach tissue [5, 6], (ii) older ‘none-omics’ studies [28], (iii) scRNAseq data were available [9, 29], or (iv) databases collated from multiple sources, e.g. Cell Marker [30] and PanglaoDB [31] (Fig. 1B and Additional file 1: Fig. S1B). Three markers were selected for each cell type, based on the following criteria: (i) a high corr. (> 0.85) between *Ref.T.* within each cell type panel (Fig. 1C and Additional file 2: Table S1, Tab 1), indicating *cell type co-expression*: parietal cells (PAC) [*ATP4B*, *MFSD4A*, *ATP4A* mean corr. ± STD 0.94 ± 0.013], chief cells (CC) [*PGC*, *LIPF*, *AZGP1*, 0.89 ± 0.013], gastric enteroendocrine cells (GEEC) [*ST18*, *INSM1*, *ARX*, 0.89 ± 0.021], gastric mucous cells (GMC) [*LGALS4*, *VILL*, *CAPN8*, 0.94 ± 0.008], mitotic cells (MTC) [*NCAPG*, *KIFC1*, *NCAPH*, 0.93 ± 0.009], endothelial cells (EC) [*PECAM1*, *CDH5*, *ERG*, 0.89 ± 0.013], fibroblasts (FB) [*PCOLCE*, *CLEC11A*, *MMP2*, 0.87 ± 0.027], macrophages (MC) [*C1QB*, *FCGR3A*, *ITGB2*, 0.86 ± 0.015], neutrophils (NP) [*CXCR2*, *FCGR3B*, *CXCR1*, 0.86 ± 0.009], T-cells (TC) [*CD3E*, *CD2*, *CD3G*, 0.9 ± 0.019], and plasma cells (PC) [*IGKC*, *JCHAIN*, *IGLC1*, 0.97 ± 0.009]; (ii) a low corr. between *Ref.T.* across the different cell type panels (Fig. 1C) (Additional file 2: Table S1, Tab 1), indicating *cell type specificity* (mean inter-panel corr. ± STD 0.08 ± 0.14); and (iii) a normal distribution of *Ref.T*. expression across the samples (Additional file 3: Fig. S2A).

#### Using reference transcript analysis to identify cell type-enriched genes

Correlation coefficients (corr.) between each selected *Ref.T.* and all other sequenced transcripts (> 56,000) were calculated across stomach RNAseq samples (Additional file 1: Fig. S1C). The proportion of cell types represented in each sample varies, due to biological and sampling variability, but ratios should remain consistent between constitutively expressed cell-enriched genes. Thus, a high corr. of a given transcript with all *Ref.T.* in only one cell type panel is consistent with enrichment in the corresponding cell type. For each cell type, a list of enriched genes was generated (Fig. 1D (i–xi)), with inclusion based on (i) the gene having a mean corr. > 0.50 with the *Ref.T.* panel representing the cell type (Additional file 1: Fig. S1C.ii) and (ii) a *differential correlation* between this value and the maximum mean corr. with any other *Ref.T.* panel > 0.15 (Additional file 1: Fig. S1D-E). This excluded genes that were potentially co-enriched in two or more cell types, as we previously described [26] (all data in Additional file 2: Table S1, Tab 2). For certain cell types, enriched genes were less well separated by corr. value that others, e.g. those most highly correlating with the fibroblast *Ref.T.* panel (Fig. 1D (vii)) tended to show elevated corr. with the *Ref.T.* panel for endothelial cells, and vice versa (Fig. 1D (vi)). However, all cell type-enriched genes were well separated when the individual gene differential correlations vs. other *Ref.T.* panels were plotted (Additional file 3: Fig. S2B), and Gene Ontology (GO) and reactome analysis [32, 33] revealed over-represented terms for these cell types were consistent with known functions e.g. for endothelial cells most significantly enriched terms included *vascular development* and *angiogenesis* (Fig. 1E.i), for fibroblasts *extracellular matrix organisation* and *collagen fibril organisation* (Fig. 1E.ii), and for T-cells *T-cell activation* and *immune response* (Fig. 1E (iii)) (Additional file 2: Table S1, Tab 8, 9 and 12). Principal component analysis of the corr. values of cell type-enriched genes [34] revealed the largest variance was between stomach-specific cell types vs. stromal/vasculature-related ones (Fig. 1F).

### Stomach cell type-enriched gene signatures

#### The majority of stomach cell type-enriched genes are protein coding

**Fig. 2:**
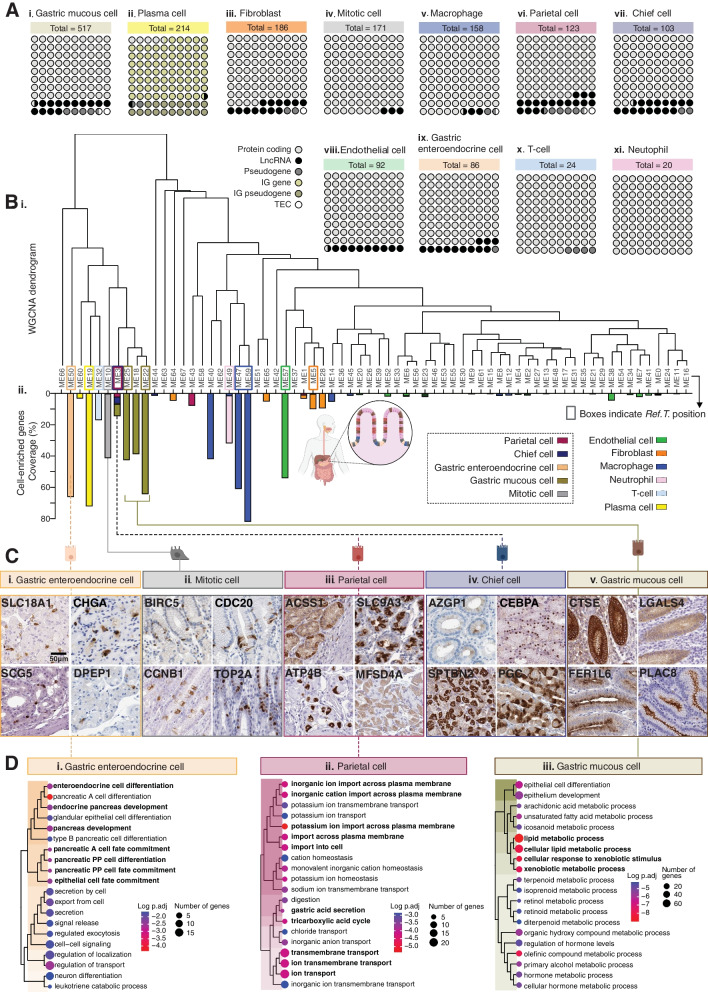
Integrative co-expression analysis of unfractionated RNAseq reveals enriched genes in human stomach cell types. (**A**) Total number and proportional representation of class for cell type enriched genes in: (i) gastric mucous cells, (ii) plasma cells, (iii) fibroblasts, (iv) mitotic cells, (v) macrophages, (vi) parietal cells, (vii) chief cells, (viii) endothelial cells, (ix) gastric enteroendocrine cells, (x) T-cells and (xi) neutrophils. (cells, (viii) endothelial cells, (ix) gastric enteroendocrine cells, (x) T-cells and (xi) neutrophils. (**B**) RNAseq data for) RNAseq data for 359 unfractionated human stomach samples was subject to weighted correlation network analysis (WGCNA). (i) Coloured squares indicate cell type Ref.T. positions on resultant dendrogram. (ii) Coloured bars show distribution of protein coding genes classified as cell type-enriched across dendrogram groups. (**C**) Human stomach tissue profiling for proteins encoded by genes classified as: (i) gastric enteroendocrine cell, (ii) mitotic cell, (iii) parietal cell, (iv) chief cell or (v) gastric mucous cell enriched. (**D**) Over-represented gene ontology terms among genes predicted to be (i) gastric enteroendocrine cell, (ii) parietal cell or (iii) gastric mucous cell enriched. See also Table S1 Tab 2, 3, 5 and 6

A total of 1694 genes were predicted to be cell type-enriched (Fig. 2A and Additional file 2: Table S1, Tab 2). Gastric mucous cells, plasma cells, and fibroblasts had the highest number of predicted enriched genes (*n* = 517, 214, and 186, respectively) (Fig. 2A (i–iii)). Of the other cell types found in all, or most, tissue types, mitotic cells, and macrophages had the most enriched genes (*n* = 171 and 158, respectively) (Fig. 1A (iv, v)). Other stomach-specialised cell types, parietal cells, chief cells, and gastric enteroendocrine cells, had significantly fewer enriched genes (*n* = 123, 103, and 86, respectively) (Fig. 2A (vi, vii, and ix), and T-cells and neutrophils had the fewest overall (*n* = 24 and 20, respectively) (Fig. 2A (x, xi)). In all cases, the majority of cell type-enriched genes were classified as protein coding [35], with the exception of plasma cells, in which the immunoglobulin (IG) gene was the most common classification (Fig. 2A (ii)). Long non-coding RNA (lncRNA) were the most common type of non-coding cell type-enriched transcript, with the exception of plasma cells, where IG pseudogene was the most common non-coding classification (Fig. 2A.ii).

### Alternative analysis and protein profiling support cell-type classifications

#### Unsupervised weighted network correlation analysis is consistent with Ref.T. analysis

As our analysis is based on manually selected *Ref.T.* panels, cell type classification is subject to an input bias. As a comparison, we subjected the same GTEx RNAseq dataset to a weighted network correlation analysis (WGCNA) [36], an unbiased method that does not require any manual input or marker gene selection. WGCNA generates corr. coefficients between all transcripts and subsequently clusters them into related groups, based on expression similarity (Fig. 2B). In general, *Ref.T*. belonging to the same cell type panel were found in the same WGCNA cluster (Fig. 2B (i), coloured boxes represent the modules in which the *Ref.T*. appeared), e.g. gastric enteroendocrine cells (cluster 50) or clusters on the same branch, e.g. gastric mucous cells (clusters 25 and 22) and macrophages (clusters 47 and 59) (Fig. 2B (i)). Protein coding genes that we predicted to be cell type enriched were predominantly clustered into the same WGCNA group as the corresponding *Ref.T.* but were also frequently classified into related modules on the same branch, consistent with our classifications (Fig. 2B (ii)). Most genes in the *Ref.T.* panels representing parietal and chief cells appeared in the same large group (cluster 3) (Fig. 2B (ii)), as were the genes in the respective predicted enriched gene lists, despite clear separation in our *Ref.T*-based method (Fig. 1C, D). Despite the lack of separation for the enriched gene signatures for parietal and chief cells by WGCNA, each contained several well-described marker genes for the respective cell type, e.g. *GIF, SLC26A7* (parietal) and *PGA4, SLC1A2* (chief cell). Indeed, we have previously shown that *Ref.T.*-based analysis can have a higher sensitivity than WGCNA for cell type gene enrichment analysis [25]. Stomach tissue protein profiling revealed staining consistent with expression in the respective cell types for proteins encoded by genes predicted to be gastric enteroendocrine cell (Fig. 2C (i)), mitotic cell (Fig. 2C (ii)), parietal cell (Fig. 2C (iii)), chief cell (Fig. 2C (iv)), or gastric mucous cell (Fig. 2C (v)) enriched. GO and reactome analysis [32, 33] revealed that over-represented terms for predicted stomach specialised cell type-enriched genes were consistent with known cell functions, e.g. for gastric enteroendocrine cells *enteroendocrine cell differentiation* (Fig. 2D (i)), for parietal cells *inorganic ion transport across the plasma membrane* and *gastric acid secretion* (Fig. 2D (ii)), and for gastric mucous cells *lipid metabolic processes* (Fig. 2D (iii)) (for all cell types see Additional file 2: Table S1, Tab 3–13).

#### Stomach cell type gene enrichment signatures

**Fig. 3:**
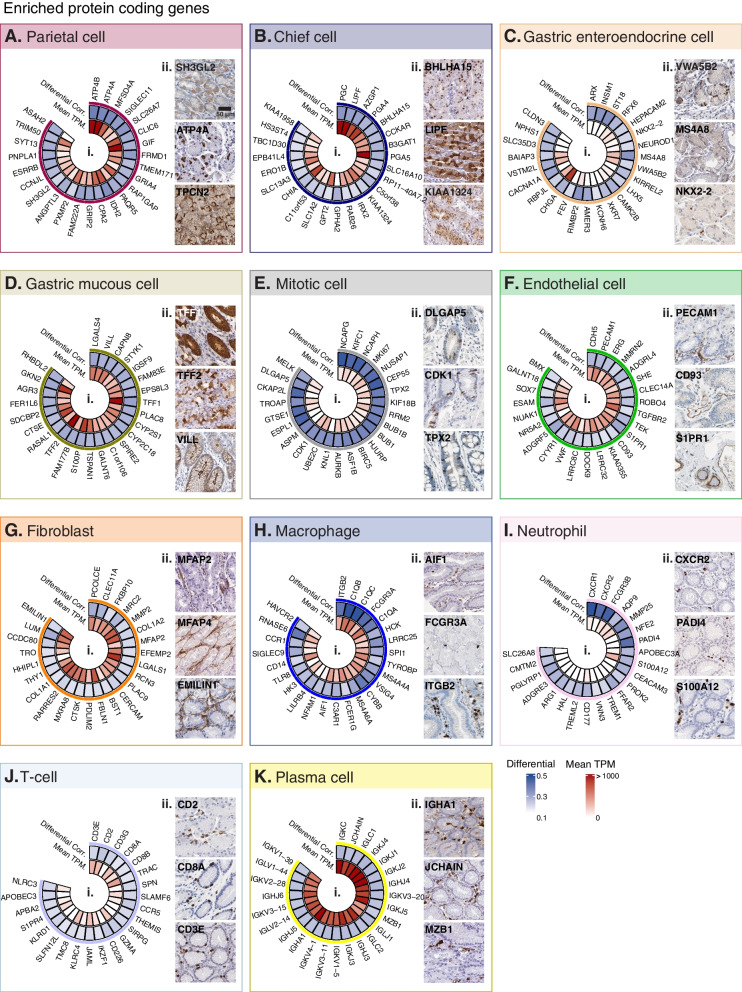
Protein coding gene signatures of human stomach cell types. Cell type-enriched protein coding genes in: (**A**) parietal cells, (**B**) chief cells, (**C**) gastric enteroendocrine cells, (**D**) gastric mucous cells, (**E**) mitotic cells, (**F**) endothelial cells, (**G**) fibroblasts, (**H**) macrophages, (**I**) neutrophils (**J**) T-cells and (**K**) plasma cells, showing: (i) differential correlation score (correlation with cell type Ref.T., panel minus max correlation with any other Ref.T. panel) and mean expression in bulk RNAseq. (ii) Human stomach tissue protein profiling for selected cell type enriched genes. See also Table S1 Tab 2

Figure 3 highlights 25 examples of enriched protein coding enriched genes for each cell type, ordered by highest corr. with the relevant *Ref.T.* panel (Fig. 3A (i)–K (i)), with differential corr. values and expression levels in the bulk RNAseq dataset (mean TPM). The mean TPM levels were generally highest for genes predicted to be enriched in parietal cells (Fig. 3A (i(), chief cells (Fig. 3B (i)), gastric mucous cells (Fig. 3D (i)), fibroblasts (Fig. 3G (i)), and plasma cells (Fig. 3K (i)) and lowest for those in mitotic cells (Fig. 3E (i)), neutrophils (Fig. 3I (i)), and T-cells (Fig. 3J (i)). This likely reflects differing numbers of each given cell type with the samples; however, as a range of expression values are observed within each given cell type, there is likely also individual gene variation in factors such as regulation and transcript stability. The highest differential values, and thus relative uniqueness among the profiled cell types, were observed for mitotic cell-enriched genes (Fig. 3E.i), most of which have well-studied roles in the regulation of the cell cycle, such as *TOP2A* and *BUB1B*. For all other cell types, top enriched genes included both known cell type-specific genes, together with those that have not been previously reported as such, e.g. *PECAM1* and *SHE* were both predicted to be endothelial cell-enriched (Fig. 3F (i)); *PECAM1* is a commonly used marker gene for this cell type, whilst there are no existing reports for the selective expression of *SHE* in this context. Tissue profiling for proteins encoded by representative cell type-enriched genes showed expression consistent with our classifications (Fig. 3A (ii)–K (ii)).

#### Ref.T. analysis can predict the source of stomach-enriched protein-coding genes

**Fig. 4:**
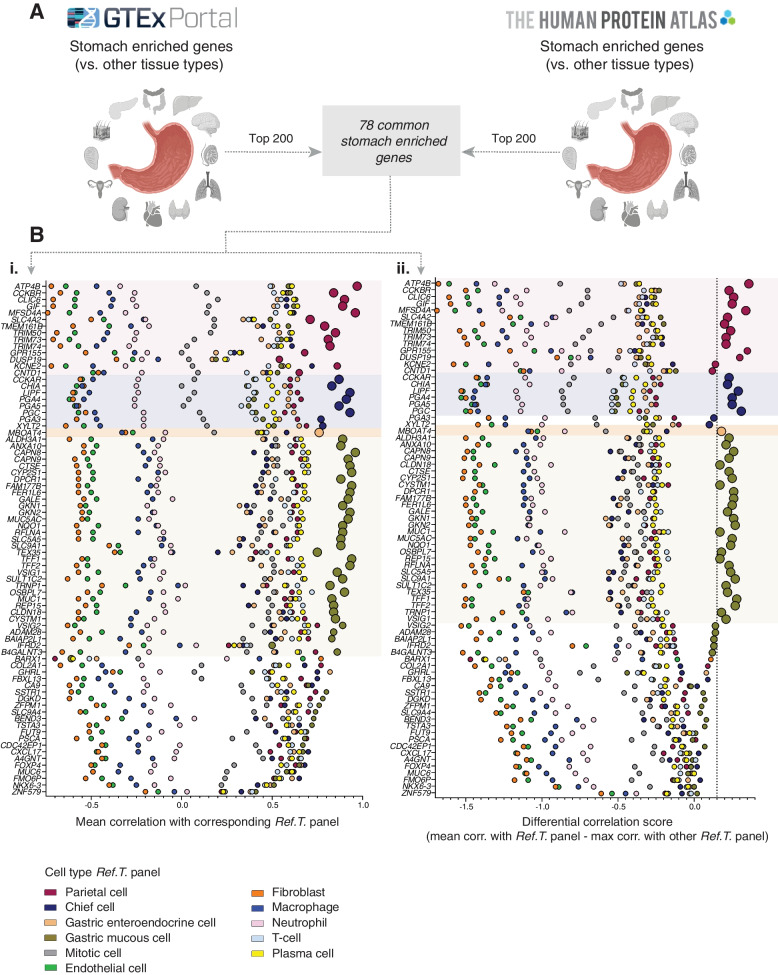
Gastric mucous cells, parietal cells and chief cells are the primary source of stomach tissue enriched genes. (**A**) The top 200 stomach enriched genes (vs. other tissue types) in RNAseq data from the GTEx Portal or Human Protein Atlas (HPA) were compared to identify genes common to both datasets (n=78). For each, the following was plotted: (**B**) (i) the mean correlation with each cell type Ref.T. panel, and (ii) the differential value vs. the next most highly correlating Ref.T. panel (dotted line indicates threshold for classification as cell type enriched). Enlarged circles represent genes with predicted cell type enrichment

Genes with enriched expression in the human stomach vs. other tissue types can be identified by a comparative analysis of unfractionated tissue RNAseq data. We extracted the top 200 human stomach-enriched genes from the HPA [6] and GTEx project [27], through the Harminozome database [37] (Fig. 4). Of the 78 genes classified as stomach-enriched in both datasets, 46/78 (59.0%) were classified as cell type enriched in our analysis, 28/46 (61.0%) in gastric mucous cells, 11/46 (24.0%) in parietal cells, 6/46 (13.0%) in chief cells, and 1/46 (2.2%) in gastric enteroendocrine cells (Fig. 4B (i, ii), respectively, large symbols). Of those not classified as cell type-enriched in our analysis (*n* = 32), 11/32 (34.4%), only narrowly failed to reach one of the thresholds for classification as either parietal-, chief-, or gastric mucous cell-enriched (Fig. 4B (i, ii), medium symbols). The majority of the remaining genes most highly correlated with *Ref.T.* panel representing one, or more, of the same cell types: parietal, chief, or gastric mucous, but were excluded from the cell type classifications due to shared enrichment. None of the stomach-enriched genes was predicted to be enriched in any cell type found across multiple tissue types, such as endothelial or immune cells, consistent with the lack of specificity of these cell types to the stomach. Thus, our analysis indicates that most stomach tissue-enriched genes are primarily expressed in gastric mucous, parietal, or chief cells.

### Cell type-enriched non-coding genes in the stomach

**Fig. 5:**
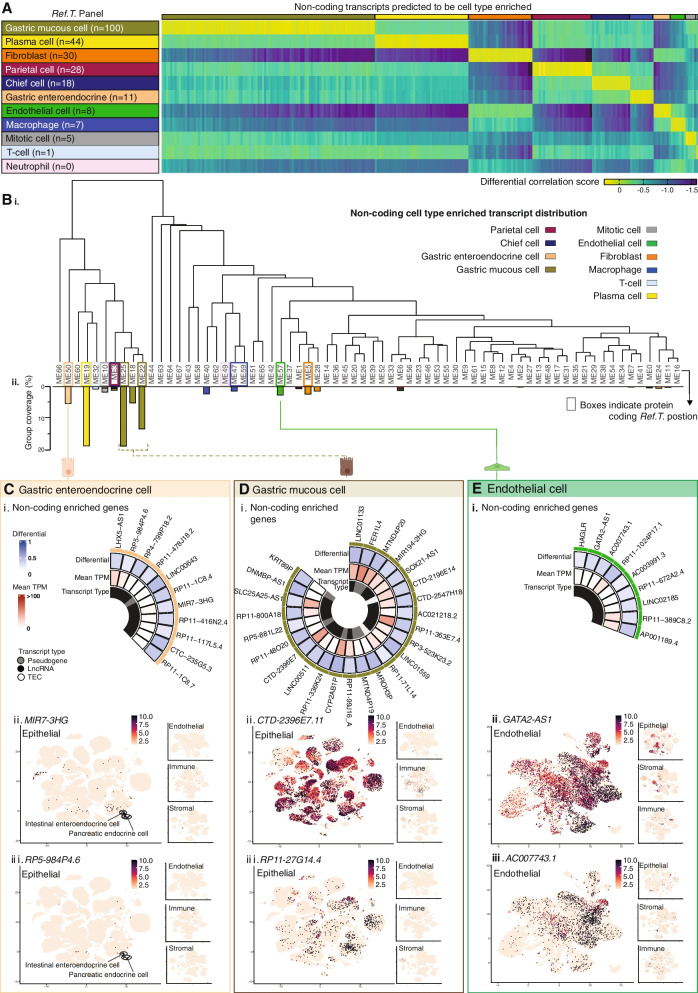
Non-coding gene signatures of human stomach cell types. (**A**) Heat map of non-coding genes predicted to be cell type enriched, showing differential score between mean correlation coefficient with the corresponding Ref.T. panel vs. highest mean correlation coefficient amongst the other Ref.T. panels. (**B**) RNAseq data for 359 unfractionated human stomach samples was subject to weighted correlation network analysis (WGCNA). (i) Coloured squares indicate cell type Ref.T. positions on resultant dendrogram. (ii) Coloured bars show distribution of non-coding genes classified as cell type-enriched across dendrogram groups. Non-coding gene enrichment signatures for: (**C**) gastric enteroendocrine cells, (**D**) gastric mucous cells and (**E**) endothelial cells, detailing: (i) up to 25 examples of cell type enriched non-coding genes, ordered by correlation coefficient with the Ref.T. panel, showing differential correlation scores (correlation with corresponding cell type Ref.T., panel minus max correlation with any other Ref.T. panel), mean expression in bulk RNAseq and transcript type. (ii and iii) scRNAseq data from analysis of epithelial, endothelial, immune or stromal cell compartments across 24 human tissues was sourced from Tabula Sapiens (Tabula Sapiens et al., [7]), and used to generate UMAP plots showing the expression profiles of example cell type enriched non-coding genes. The largest plot shows the compartment with the highest expression. See also Table S1 Tab 2 and Figure S3 (for all UMAP plot annotations)

**Fig. 6:**
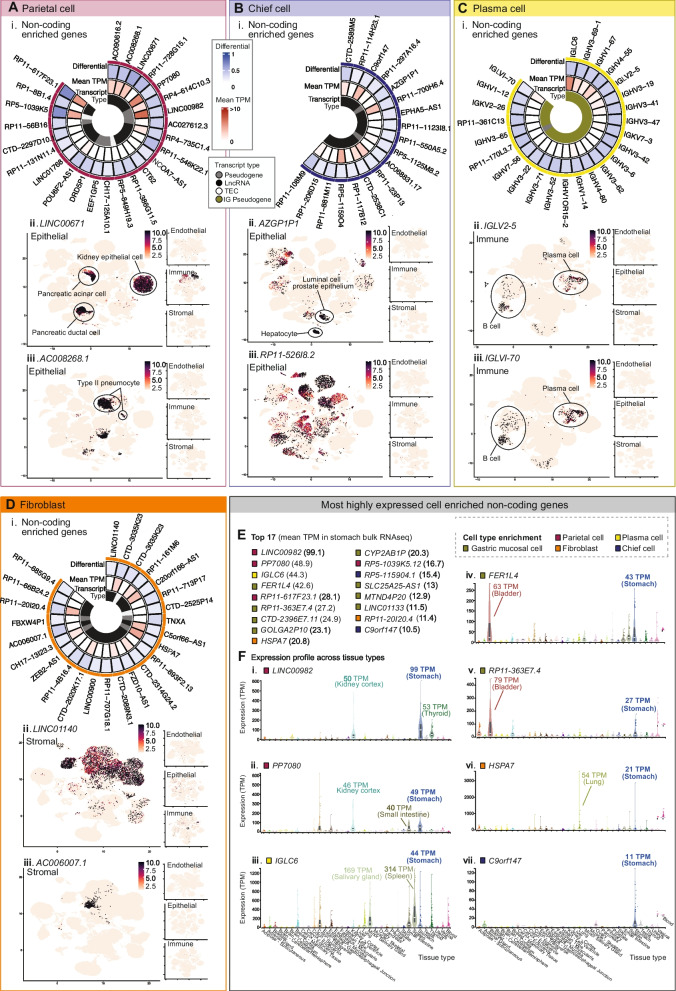
Core non-coding gene signatures of human stomach cell types and tissue distribution patterns. Non-coding gene enrichment signatures for: (**A**) parietal cells, (**B**) chief cells, (**C**) plasma cells and (**D**) endothelial cells, detailing (i) up to 25 examples of cell type enriched non-coding genes, ordered by correlation coefficient with the Ref.T. panel, showing differential correlation scores (correlation with corresponding cell type Ref.T., panel minus max correlation with any other Ref.T. panel), mean expression in bulk RNAseq and gene type. (ii and iii) scRNAseq data from analysis of epithelial, endothelial, immune, or stromal cell compartments across 24 human tissues was sourced from Tabula Sapiens (Tabula Sapiens et al., 2022), and used to generate UMAP plots showing the expression profiles of example cell type enriched non-coding genes. The largest plot shows the compartment with the highest expression. (**E**) The most highly expressed cell type enriched non‐coding genes in stomach bulk RNAseq. (**F**) Expression of genes classified as enriched in parietal cells: (i) LINC00982 and (ii) PP7080, plasma cells: (iii) IGLC6, gastric mucous cells: (vi) FER1L4 and (v) RP11-363E7.4, fibroblasts: (vi) HSPA7 and chief cells: (vii) C9orf147, in bulk RNAseq of different human organs. Mean TMP expression is annotated for selected organs on each plot. See also Table S1 Tab 2 and Figure S2 (for all UMAP plot annotations)

A total of 252 non-coding genes were identified as cell type-enriched in the stomach (Fig. 5A), the greatest number of which were in gastric mucous cells, plasma cells, or fibroblasts (*n* = 100, 44, and 30, respectively). When the sample set was analysed by WGCNA (Fig. 5B (i)), non-coding genes that we predicted to be cell type enriched predominantly clustered into the same WGCNA group as the corresponding *Ref.T.*, or into adjacent groups on the same branch (Fig. 5B (ii)). Up to 25 examples of non-coding enriched genes in gastric enteroendocrine cells (Fig. 5C (i)), gastric mucous cells (Fig. 5D (i)), endothelial cells (Fig. 5E (i)), parietal cells (Fig. 6A (i)), chief cells (Fig. 6B (i)), plasma cells (Fig. 6C (i)), and fibroblasts (Fig. 6D (i)), ordered by corr. with the relevant *Ref.T* panel, are displayed with differential corr. values vs. other profiled cell types, expression in the bulk RNAseq data (mean TPM), and transcript type. In all cell types, with the exception of plasma cells, where the most common type of enriched non-coding gene was IG pseudogene (Fig. 6C (i)), long non-coding RNAs made up the majority of the predicted enriched genes. Generally, gastric mucous cell (Fig. 5D (i)) and fibroblast (Fig. 6D (i)) enriched non-coding genes were expressed at the highest levels in the stomach bulk RNAseq. This likely reflects the differing numbers of each given cell type within the samples, but the intra-cell type variation also indicates individual gene regulation.

There is currently no existing dataset of non-coding enriched genes in stomach cell types that could be used to validate our predictions. However, we sourced scRNAseq data from the analysis of 24 tissue types in Tabula Sapiens [7] (data for the stomach was not available) that had been classified into endothelial, epithelial, immune, and stromal cell functional compartments (for Tabula Sapiens UMAP cell type classifications, see Additional file 4: Fig. S3 A-D). We generated UMAP plots for each of these compartments to determine expression profiles for selected non-coding genes that we predicted to be cell type enriched. The predicted gastric enteroendocrine enriched genes *MIR7-3HG* and *RP5-984P4.6* were expressed only in the epithelial cell compartment, specifically in the clusters annotated as intestinal enteroendocrine and pancreatic alpha and beta cells (Fig. 5C (ii, iii)), consistent with a specialised role in endocrine cells, not only in the stomach, but also in the pancreas and other parts of the GI tract. The predicted gastric mucous cell-enriched genes *CTD-2396E7.11* and *RP11-27G14.4* were widely expressed in the epithelial compartment but not in the endothelial, immune, or stromal cell compartments (Fig. 5D (ii, iii)). The predicted endothelial cell-enriched genes *GATA2-AS1* and *AC007743.1* were expressed predominantly in the endothelial cell compartment (Fig. 5E (ii, iii)), also consistent with our classifications. Genes predicted to be parietal cell enriched, *LINC00671* and *AC008268.1* (Fig. 6A (ii, iii)), and chief cell enriched, *RP11-526I8.2* and *AZGP1P1* (Fig. 6B (ii, iii)), were predominantly expressed in the epithelial compartment. The type of epithelial cell in which the genes were expressed varied, e.g. the chief cell enriched gene *AZGP1P1* (Fig. 6B (ii)) was expressed predominantly in luminal cells of the prostate and hepatocytes; one could speculate that this gene indicates a shared secretory function between these specific cell types, whilst *RP11-526I8.2* was more generally expressed in the epithelial compartment (Fig. 6B (iii)) perhaps indicating a more general role. The predicted plasma cell-enriched genes *IGLV2-5* and *IGLVI-70* were expressed only in the immune cell compartment (Fig. 6C (ii, iii)) in clusters annotated as either plasma cells or B-cells. The predicted fibroblast-enriched genes *LINC01140* and *AC006007.1* were expressed predominantly in the stromal cell compartment (Fig. 6D (ii, iii)), also consistent with our classifications. Thus, the Tabula Sapiens scRNAseq data provides supportive evidence for our cell type classifications, despite the lack of stomach cell type analysis in this dataset.

Of those non-coding genes that we classified as cell type enriched, 17 had relatively high expression in the bulk RNAseq stomach samples (mean TPM > 10) and were most frequently predicted to be gastric mucous cell enriched (Fig. 6E). To determine the expression profile of these genes in different organ types, we sourced data from bulk RNAseq of other tissues in GTEx (Fig. 6F). The most highly expressed parietal cell enriched non-coding genes, *LINC00982* and *PP7080* (mean TPM 99 and 49, respectively), both had high relative expression in stomach tissue (Fig. 6F (i, ii)), consistent with a specialised function in this organ. *IGLC6*, the most highly expressed non-coding transcript we predicted to be enriched in plasma cells, was highly expressed in the spleen and salivary gland: tissues that contain high numbers of plasma cells (Fig. 6F (iii)). The most highly expressed non-coding genes we predicted to be enriched in gastric mucous cells, *FER1L4* and *RP11-363E7.4*, both had high relative expression in the stomach and bladder (Fig. 6F (iv, v)); one could speculate these genes have specific functions in the mucous cells found in these tissue types. *HSPA7*, the most highly expressed predicted fibroblast-enriched gene had variable expression across tissue types (Fig. 6F (vi)), consistent with the ubiquitous presence of this cell type across organs, whilst the chief cell enriched transcript, *C9orf147*, had high relative expression only in stomach tissue (Fig. 6F (vii)). Thus, the most highly expressed non-coding genes predicted to be enriched in the stomach specialised cell types were detected at relatively high levels in stomach tissue (and in relatively few other tissue types), consistent with a specialised function here. Conversely, those predicted to be enriched in less specialised cell types, such as plasma cells, were more broadly expressed across tissue types, consistent with a common cell type function in multiple organs. All data for non-coding genes can be searched via the web portal https://cell-enrichment.shinyapps.io/noncoding_stomach/.

#### Stomach tissue scRNAseq supports Ref.T. analysis

To our knowledge, there is no existing comprehensive scRNAseq dataset where all healthy stomach cell types have been analysed and compared, to use for further confirmation of our findings. However, we performed a comparison between our results and two stomach RNAseq studies, one with a focus on the analysis of stomach epithelial cell types [9] and another where cell types from gastric mucosa samples in premalignant and early-malignant lesions were characterized [11] (Additional file 2: Table S1, Tab 14). Neither study contained all cell types we profiled and for some cell types, such as gastric enteroendocrine cells, classification and/or terminology varied, as is typical [38]. Therefore, we made comparisons between closely related cells or cell sub-types across studies, e.g. those annotated as D cells, G cells, X cells, antral enterochromaffin cells, and oxyntic enterochromaffin-like cells in Busslinger et. al. [9] were considered together as a single group and compared to data for ‘enteroendocrine cells’ in the current study, and that by Zhang et. al. [11]. Where data was available, we also compared our results to those from our HPA single cell section [29] and to cell type marker genes defined by the analysis of multiple human and murine datasets [31] (Additional file 2: Table S1, Tab 14 [row 2 states source and cell type annotation]). Independent verification of genes we classified as cell type enriched (i.e. those that had a comparable classification in at least one other independent dataset) varied between cell types (Additional file 2: Table S1, Tab 14). Macrophages and T-cells had the highest agreement with one or more of the independent studies (71 and 96%, respectively). As expected, cell types with a higher proportion of non-coding and/or lowly expressed predicted enriched genes, such as gastric mucous cells, fibroblasts, and parietal cells (the latter of which were also only represented in one study) tended to have a lower level of independent validation (40%, 38%, and 30%, respectively). However, Gene Ontology and reactome analysis of genes that were *not* validated in the other datasets were significantly enriched for terms linked to the predicted cell type, e.g. for gastric mucous cells *lipid metabolic processes* (adjusted FDR 1.9 × 10^−03^), for fibroblasts *extracellular structure organisation* (adjusted FDR 3.8 × 10^−02^), and for parietal cells *monoatomic ion transport* (adjusted FDR 2.4 × 10^−03^) and *gastric acid secretion* (adjusted FDR 1.1 × 10^−02^), consistent with these genes having specialised roles in these cell types.

To compare global cell profiles from the stomach RNAseq datasets [9, 11] with our study and each other, we calculated the significance of the overlap between cell type-enriched genes, using a hypergeometric test (Additional file 4: Fig. S3 E). Genes predicted to be cell type enriched in our study were significantly overrepresented in enriched genes in the corresponding cell types in both scRNAseq studies (Additional file 4: Fig. S3 E). This overlap was comparable to, or more significant than, that between the two scRNAseq studies themselves. Whilst such comparisons can be helpful, the variability between these studies (i.e. general vs. specific cell type focus, healthy vs. diseased tissue) means the data can only be used as supported evidence, as opposed to a definitive validation.

#### Comparison of predicted sex-specific stomach cell type-enriched genes

**Fig. 7:**
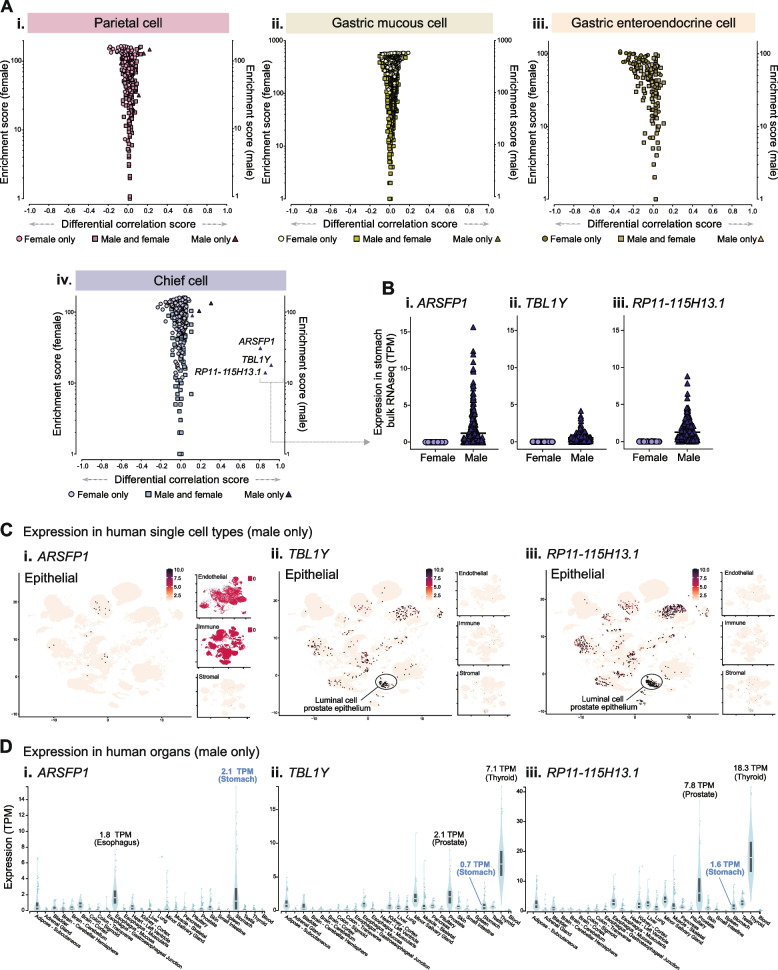
Identification of sex-specific cell-enriched genes in human stomach tissue. (**A**) Human stomach RNAseq data (*n*=359 individuals) was retrieved from GTEx V8 and divided into female (*n*=132) and male (*n*=227) subgroups before classification of cell type-enriched genes. For genes classified as: (i) parietal, (ii) gastric mucous, (iii) gastric enteroendocrine or (vi) chief cell enriched in either sex, the ´sex differential corr. score’ (difference between mean corr. with the Ref.T. panel in females vs. males) was plotted vs. ‘enrichment score´ (position in each respective enriched list, highest score = highest corr.). On each plot, genes enriched in both females and males are represented by common-coloured square symbols, and genes classified as enriched only in females or males are represented by differently coloured circle and triangle symbols, respectively. (**B**) Expression in female or male samples for genes classified as male-only enriched in chief cells: (i) ARSFP1, (iii) TBL1Y and (iii) RP11-115H13.1. (**C**) scRNAseq data from analysis of epithelial, endothelial, immune or stromal cell compartments across human tissues from male donors was sourced from Tabula Sapiens (Tabula Sapiens et al., [7]), and used to generate UMAP plots showing the expression profiles of: (i) ARSFP1, (iii) TBL1Y and (iii) RP11-115H13.1. (**D**) Expression of: (i) ARSFP1, (iii) TBL1Y and (iii) RP11-115H13.1 in bulk RNAseq of different human organs from male donors. The largest plot shows the compartment with the highest expression. Mean expression is annotated for selected organs on each plot. See also Table S2 Tab 1, Figure S2 (for all UMAP plot annotations) and Figure S3

We performed a subset analysis of the stomach RNAseq dataset (male *n* = 227, female *n* = 132,), to identify sex-specific cell type-enriched genes. Similar to the full dataset, intra-panel cell type *Ref.T.* correlated well in single-sex sample subsets (all > 0.84) (Additional file 5: Table S2, Tab 1, Table A and B). Cell type-enriched genes were calculated for the whole dataset. To compare gene enrichment profiles in males and females, the following was calculated for any gene that was classified as cell type enriched in either subset: (i) the *differential correlation score*, defined as the difference between the mean corr. coefficient with the cell type *Ref.T*, in the male and female sample subsets (to highlight potential differences in enrichment between the sexes) and (ii) the *enrichment score*, based on the mean corr. value with the *Ref.T.* panel (*highest score* = highest corr.) (to give an overview of the relative degree of enrichment of highlighted genes). Cell profiles were mainly comparable between sexes, for both stomach-specialised cell types (Fig. 7A (i–iv)) and others (Additional file 6: Fig. S4 A-G) (genes enriched in *both* males and females represented by square symbols). For those genes classified as enriched *only* in males or females (represented by differently coloured triangle and circle symbols, respectively), most had differential corr. scores close to 0; indicating that they fell marginally below the designated threshold for classification as enriched in the other sex. A small number of distinct male-only enriched genes were identified in chief cells: *ARSFP1*, *TBL1Y*, and *RP11-115H13.1* (Fig. 7A (iv)), all of which were Y-linked, with expression levels above background level only in male samples (Fig. 7B (i–iii)). As described above, we sourced scRNAseq data from Tabula Sapiens [7] for cells classified as endothelial, epithelial, immune, or stromal (Additional file 4: Fig. S3 A-D). We generated UMAP plots (using cell data from male donors only) to show the expression profiles of the male-only chief cell-enriched genes. *ARSFP1* was detected only at low levels in the epithelial compartment (Fig. 7C (i)), whilst *TBL1Y* (Fig. 7C (ii)) and *RP11-115H13.1* (Fig. 7C (iii)) had strikingly similar expression profiles, with the highest levels in both cases detected in prostate epithelial cells. All three male-only chief cell-enrichened genes had low/no expression in the endothelial, immune, or stromal compartments (Fig. 7C (i–iii)). To determine the broad expression profile of the most highly expressed non-coding enriched genes across organs (from male donors), we sourced data from GTEx (Fig. 7D). *ARSFP1* had enhanced expression only in the stomach and oesophagus (Fig. 7D (i)); both of which are tissue types not included in the Tabula Sapiens dataset, consistent with the low detection observed there. *TBL1Y* and *RP11-115H13.1* had similar expression profiles across tissue types, with enhanced expression in the thyroid (which was also absent from the Tabula Sapiens dataset) followed by the prostate, in keeping with the high expression observed in prostate epithelial cells in the scRNAseq (Fig. 7D (ii, iii)). Thus, one could speculate that male-only chief cell-enriched gene *ARSFP1* has a stomach-specific function, whilst *TBL1Y* and *RP11-115H13.1* appear to be co-expressed also in cell types outside the stomach, suggesting a broader function in multiple cell types.

## Discussion

Here, we present a genome-wide cell type-enriched transcriptome atlas for the human stomach, using our previously described method to resolve unfractionated tissue RNAseq data to the cell type level [24–26]. Our method circumvents some challenges associated with scRNAseq analysis, including issues associated with cell isolation, material amplification [18, 39, 40], and induction of expression artefacts, due to loss of tissue-specific cues or processing [15]. Our analysis incorporates a high number of biological replicates, reducing the impact of individual variation and allowing for well-powered subgroup comparisons, e.g. female vs. male. As data for gene enrichment signatures of stomach cell types are lacking in the existing literature, with this organ absent from large-scale scRNAseq initiatives, such as Tabula Sapiens [7] and the Human Cell Atlas [8], our study provides a useful resource, which can be searched on a gene-by-gene basis on the HPA (www.proteinatlas.org/humanproteome/tissue+cell+type/stomach) or https://cell-enrichment.shinyapps.io/noncoding_stomach/, for protein coding and non-coding genes, respectively.

Of the 11 cell types we profiled in the stomach, gastric mucous cells had the highest number of predicted enriched genes, which included those encoding for proteins with known cell type-specific functions, such as in mucosal defence, e.g. *CAPN8*, *CAPN9* [41], *GKN1* [42], *MUC13* [43], *TFF1*, and *TFF2* [44], and lipid metabolism, e.g. *PLPP2* [45], *PPARG* [46], and *PLA2G10* [47]. In addition, several genes we identified have no reported role in this cell type, including *FAM83E*, *CYP2S1*, and *PLAC8*. It was not possible to identify discrete expression signatures for gastric mucous cell subsets, i.e. MUC6 + gland mucous cells vs. MUC5AC + pit mucous cells. As MUC5AC was classified as strongly gastric mucous cell enriched in our analysis, one could speculate that the profiled population is primarily composed of pit mucous cells, which is consistent with the higher prevalence of this sub-type in the region of the stomach (body) that was analysed [48].

Predicted gastric enteroendocrine-enriched genes also included those with known cell type function, such as *CAMK2B*, which is involved in intracellular calcium signalling [49], and the neuroendocrine secretory protein *CHGA* [50]. Other predicted gastric enteroendocrine enriched genes had not been described in gastric enteroendocrine cells previously, such as *LHX5*, *SERPINA10*, and *KCNH6*. *LHX5* has mainly been studied in the context of neuronal development [51, 52], but in the GTEx database, the only tissue type, outside the brain, where *LHX5* had elevated expression compared to others was the stomach [27]; thus, one could speculate that this gene also has a specific functional role here. *SERPINA10* was previously identified as a biomarker for gastrointestinal neuroendocrine carcinoma [53], and *KCNH6* has a role in the regulation of insulin secretion in the pancreas [54]; both were consistent with our prediction that these genes have an endocrine cell enriched profile.

Many genes we predicted to be parietal cell enriched were well-known markers of this cell type, such as *GIF* [55] and *SLC26A7* [56]. However, others had no reported cell type-specific expression or function, such as *ACSS1*, a mitochondrial matrix protein functioning as a catalyst of acetyl-CoA synthesis [57] and *MFSD4*, a marker for hepatic metastasis in gastric cancer [58]. Our classifications were supported by a scRNAseq study that showed elevated expression of *ACSS1* and *MFSD4* in parietal cells vs. other stomach epithelial cells [9]. Other predicted enriched genes for which a function in parietal cells has not yet been described included *SLC12A3*, *ETNPPL*, *FNDC10*, *TUBA3C*, *TRIM73*, *TRIM74*, and *CLCNKA*. Chief cell-enriched genes included *BHLHA15*, a known chief cell marker [59], and *KIAA1324*, which is required for chief cell secretory granule maturation [60]. Novel predicted chief cell-enriched genes included the orphan receptor *GPR150*, a G-protein coupled receptor in which aberrant methylation has been linked to ovarian cancer [61]; *MOGAT1*, a monoacylglycerol acyltransferase that functions in the absorption of dietary fat in the intestine [62]; and *LIPK*, previously identified in the epidermis with a function in lipid metabolism [63].

Whilst there is no existing database of non-coding gene enrichment profiles in the cell types of the stomach, and a lack of information regarding the function of any such genes in normal tissue, increasing evidence of the involvement of non-coding genes in the development of gastric cancer [22, 64, 65] and associated drug resistance [66] indicates that this transcript class has important functions in this tissue type. Of the stomach-specialised cell types we profiled, gastric mucous cells had the highest number of predicted enriched non-coding genes, which included several antisense transcripts to corresponding gastric mucous cell-enriched protein-coding genes, such as *SOX21-AS1* and *TRIM31-AS1*, suggesting a local regulation of gene transcription. Many gastric mucous cell enriched non-coding genes were expressed at relatively high levels, compared to other non-coding genes in the same or other cell types, including *LINC01133*, *FER1L4*, *RP11-363E7.4*, and *CTD-2396E7.11*. *LINC01133* and the pseudogene *FER1L4* are inhibitors of gastric cancer progression, with reduced expression associated with a more aggressive tumour phenotype [67, 68]. To date, there is a single publication on *RP11-363E7.4*, where a genome-wide screen of gastric cancer samples identified it as a key regulator of disease progression, with higher expression associated with overall survival [69]. All the aforementioned studies were based on analysis of bulk RNAseq cancer samples, and the cell type in which these genes primarily function in healthy tissue is not reported; our data strongly indicates that this site is the mucous cell compartment. *CTD-2396E7.11* has not been described in the context of gastric cancer, but it was identified as one of four hub lncRNAs associated with reduced colon adenocarcinoma progression [70]. As this tumour type also arises from the mucosa, one could speculate *CTD-2396E7.11* has a similar expression profile in healthy colon tissue. *LIN00982*, the highest expressed of all classified non-coding genes, was enriched in parietal cells and had, similar to those discussed above been shown to have a role in the inhibition of gastric cancer progression [71].

Examples of non-coding genes we predicted to have gastric enteroendocrine cell-enriched expression included *MIR7-3HG* and *RP5-984P4.6*. The selective expression of these genes in pancreatic and intestinal endocrine cells [7] is consistent with them having a conserved endocrine function. *MIR7-3HG* can act as an autophagy inhibitor [72], but there are no reports of its function in an endocrine context. *RP5-984P4.6* is currently completely uncharacterised. Other gastric enteroendocrine cell enriched non-coding genes included *LHX5-AS1*, an antisense transcript to the gastric enteroendocrine cell enriched corresponding protein-coding gene.

Despite reported differences in stomach function between males and females, such as in speed of gastric emptying [73], gastrointestinal motility [74], incidence of gastric cancer [75], and gastric cancer survival [76], there are no studies of sex differences between stomach cell-type gene enrichment profiles. We found that global cell type gene enrichment signatures were similar between sexes, but we did identify 3 male-only chief cell-enriched genes—*ARSFP1*, *RP11-115H13.1*, and *TBL1Y*, all of which were Y-linked [77, 78]. In the GTEx database, the pseudogene *ARSFP1* was most highly expressed in male stomach samples, compared to the other 53 tissue types profiled from males [27], supportive of a currently unknown sex and tissue-specific role, and consistent with our predicted enrichment in a stomach-specific cell type in males. Although it is often assumed that pseudogenes lack function, recent studies have shown that they can have key roles, functioning as antisense, interference or competing endogenous transcripts [79–81]. *RP11-115H13.1* was one of only eight lncRNAs identified as associated with a high risk of gastric cancer [82], but the dataset analysed in this study contained both male and female samples, meaning the prognostic value of *RP11-115H13.1* in male patients was likely underestimated. To our knowledge, there are no existing reports of the potential cellular function of *RP11-115H13.1* or *ARSFP1*. *TBL1Y* has been reported as involved in syndromic hearing loss [83] and cardiac differentiation [84], but studies of its function in the stomach are lacking.

There are limitations in our study. The RNAseq data we analysed is generated from samples taken from the corpus (body) of the stomach, so specialised cell profiles found in other regions of the stomach may not be represented in our dataset. We do not profile cell subtypes, such as those included under the umbrella term of ‘gastric enteroendocrine cells’ including D-cells and G-cells, for which it was not possible to identify *Ref.T.* that fulfilled the required criteria. Our observations are consistent with these sub-cell types being typically defined by the expression of a limited number of specialised proteins [85–87], rather than large distinct gene signature panels. Gene expression in the stomach can be modified by genetic or environmental factors, such as the individual variation in the gastrointestinal microbiome [88]. Strongly regulated genes may therefore not correlate with the more constitutively expressed *Ref.T.* selected to represent the cell type in which they are primarily expressed, as variation across samples could be independent of cell type proportions. Thus, such genes could be false negatives in our analysis. Furthermore, we have used high thresholds for the classification of genes as cell type-enriched, which could lead to incorrect exclusion. For example, tissue profiling showed that proteins encoded by *MUC4* and *MUC5B* are selectively expressed in gastric mucous cells [89], but they fall just below the threshold for classification as such in our analysis. In addition, the exclusion of lowly expressed genes from the analysis may also result in false-negative classifications for rarer cell types, for example, *PAX6*, which controls endocrine cell differentiation [90], and proglucagon [91] and gastric inhibitory polypeptide [92] production, was excluded from classification as a gastric enteroendocrine enriched gene only due to expression level below the designated cut off. However, in all cases the individual enrichment scores clearly indicate a cell-type enriched expression; thus, our classifications should be regarded as a guide, and the data should be considered on a gene-by-gene basis.

## Conclusions

Here, we present a genome-wide cell type-enriched transcriptome atlas for the human stomach and provide an open access database for the research community.

## Methods

### Lead contact

Further information and requests for resources and reagents should be directed to and will be fulfilled by the lead contact: Dr. Lynn Marie Butler (email: Lynn.butler@ki.se).

### Experimental model and subject details

Bulk RNAseq data analysed in this study was obtained from the Genotype-Tissue Expression (GTEx) Project (gtexportal.org) [27] accessed on 2021/04/26 (dbGaP Accession phs000424.v8.p2). Transcript types were categorised according to Biotype definitions in ENSEMBL release 102 [35]. Human tissue protein profiling was performed in-house as part of the HPA project [6, 93, 94] (www.proteinatlas.org). Human stomach tissue samples were obtained from the Department of Pathology, Uppsala University Hospital, Uppsala, Sweden, as part of the Uppsala Biobank. Samples were handled in accordance with Swedish laws and regulations, with approval from the Uppsala Ethical Review Board [6].

### Method details

#### Tissue profiling: human tissue sections

Stomach tissue sections were stained, as previously described [6, 93]. Briefly, formalin-fixed and paraffin-embedded tissue samples were sectioned, de-paraffinised in xylene, hydrated in graded alcohols, and blocked for endogenous peroxidase in 0.3% hydrogen peroxide diluted in 95% ethanol. For antigen retrieval, a Decloaking chamber® (Biocare Medical, CA) was used. Slides were boiled in Citrate buffer®, pH6 (Lab Vision, CA). Primary antibodies and a dextran polymer visualisation system (UltraVision LP HRP polymer®, Lab Vision) were incubated for 30 min each at room temperature, and slides were developed for 10 min using Diaminobenzidine (Lab Vision) as the chromogen. Slides were counterstained in Mayers haematoxylin (Histolab) and scanned using Scanscope XT (Aperio). Primary antibodies, source, target, and identifier are as follows: atlas antibodies: ACSS1 (Cat#HPA043228, RRID:AB_2678372), ATP4A (Cat#HPA076684, RRID:AB_10672772), ATP4B (Cat#HPA045400, RRID:AB_2679314), MFSD4A (Cat#055407), SH3GL2 (Cat#HPA026685, RRID:AB_1856817), SLC9A3 (Cat#HPA036493, RRID:AB_10673353), TPCN2 (Cat#HPA027080, RRID:AB_10600917), CEBPA (Cat#HPA065037, RRID:AB_2685410), LIPF (Cat#HPA045930, RRID:AB_10959518), SPTBN2 (Cat#HPA043529, RRID:AB_2678531), BHLHA15 (Cat#HPA047834, RRID:AB_2680172), KIAA1324 (Cat#HPA029869, RRID:AB_10794320), PGC (Cat#HPA031717, RRID:AB_10670130), CAMK2B (Cat#HPA053973, RRID:AB_2682328), SLC18A1 (Cat#HPA063797, RRID:AB_2685125), MS4A8 (Cat#HPA007319, RRID:AB_1854138), NKX2-2 (Cat#HPA003468, RRID:AB_1079490), TFF2 (Cat#HPA036705, RRID:AB_2675263), VILL (Cat#HPA035675, RRID:AB_10671223), CTSE (Cat#HPA012940, RRID:AB_2668773), FER1L6 (Cat#HPA054117, RRID:AB_2682387), LGALS4 (Cat#HPA031186, RRID:AB_2673778), PLAC8 (Cat#HPA040465, RRID:AB_10794875), CCNB1 (Cat#HPA061448, RRID:AB_2684522), DLGAP5 (Cat#HPA005546, RRID:AB_1078677), TPX2 (Cat#HPA005487, RRID:AB_1858223), PECAM1 (Cat#HPA004690, RRID:AB_1078462), CD93 (Cat#HPA009300, RRID:AB_1846342), MFAP2 (Cat#HPA007354, RRID:AB_1079365), MFAP4 (Cat#HPA054097, RRID:AB_2682378) EMILIN1 (Cat#HPA002822, RRID:AB_1078738), AIF1 (Cat#HPA049234, RRID:AB_2680685), ITGB2 (Cat#HPA016894, RRID:AB_1846257), CXCR2 (Cat#HPA032017, RRID:AB_2674112), PADI4 (Cat#HPA017007, RRID:AB_1854921), S100A12 (Cat#HPA002881, RRID:AB_1848175), CD2 (Cat#HPA003883, RRID:AB_1846263), CD3E (Cat#HPA043955, RRID:AB_2678747), IGHA1 (Cat#HPA001217, RRID:AB_1079120), JCHAIN (Cat#HPA044132, RRID:AB_2678826) and MZB1 (Cat#HPA043745, RRID:AB_10960359) SCG5 (Cat#HPA013136, RRID:AB_1856657), DPEP1 (Cat#HPA01278, RRID:AB_1847842), VWA5B2 (atlas antibodies Cat#HPA036823, RRID:AB_10672269), from Santa Cruz Biotechnology: AZGP1 (Cat#sc-13585, RRID:AB_667849), BIRC5 (Cat#sc-17779, RRID:AB_628302), CDC20 (Cat#sc-13162, RRID:AB_628089), S1PR1 (Cat#sc-48356, RRID:AB_2238920), FCGR3A (Cat#sc-20052, RRID:AB_626925) from Agilent: CD8A (Cat#M7103, RRID:AB_2075537) from Leica Biosystems: TOP2A (Cat#NCL-TOPOIIA, RRID:AB_564035), TFF1 (Cat#NCL-pS2, RRID:AB_563985) from Epitomics an AbCam company: CDK1 (Cat#1161–1, RRID:AB_344898) and from Roche: CHGA (Product name: 1199 021).

### Quantification and statistical analysis

#### Reference transcript-based correlation analysis and criteria for cell type enrichment

This method was adapted and expanded from that previously developed to determine the cross-tissue pan-EC-enriched transcriptome [24] and human brain and adipose tissue cell-enriched genes [25, 26].

Human stomach bulk RNAseq data (*N* = 359) was downloaded from the Genotype-Tissue Expression (GTEx) portal V8 (https://gtexportal.org). Analysed samples were collected from the corpus (body) of the stomach, and donor age groups were represented as follows: 2029 years *n* = 44, 30–39 years *n* = 39, 40–49 years *n* = 64, 50–59 years *n* = 128, and 60–70 years *n* = 84. Pairwise Spearman correlation coefficients were calculated between reference transcripts selected as proxy markers (‘*Ref.T.* panels’) for parietal cells [*ATP4B*, *MFSD4A*, *ATP4A*], chief cells [*PGC*, *LIPF*, *AZGP1*], gastric enteroendocrine cells [*ST18*, *INSM1*, *ARX*], gastric mucous cells [*LGALS4*, *VILL*, *CAPN8*], mitotic cells [*NCAPG*, *KIFC1*, *NCAPH*], endothelial cells [PECAM1, CDH5, ERG], fibroblasts [*PCOLCE*, *CLEC11A*, *MMP2*], macrophages [*C1QB*, *FCGR3A*, *ITGB2*], neutrophils [*CXCR2*, *FCGR3B*, *CXCR1*], T-cells [*CD3E*, *CD2*, *CD3G*], and plasma cells [*IGKC*, *JCHAIN*, *IGLC1*] and all other sequenced transcripts. Correlation coefficients were calculated in R using the *corr.test* function from the *psych* package (v 1.8.4) and false discovery rate (FDR) adjusted *p*-values (using Bonferroni correction), and raw *p*-values were calculated. Genes were classified as cell type enriched when the following criteria were fulfilled: (i) a mean correlation > 0.50 (FDR < 0.0001) with the *Ref.T.* panel representing that cell type, (ii) a minimum ‘differential correlation’ between this value and the *next highest* mean correlation with any other *Ref.T.* panel (representing another cell type) > 0.15, and (iii) TPM expression < 0.1 in over 50% of samples. See Additional file 1: Fig. S1 for the method overview.

#### Weighted correlation network (WGCNA) analysis

The R package WGCNA [36] was used to perform co-expression network analysis for gene clustering, on log2 expression TPM values. Transcripts with a TPM = 0 in > 50% of samples were excluded prior to WGCNA analysis, leaving 28,254 gene transcripts for analysis. The soft threshold power was chosen based on the scale-free topology index and was set at 19; clustering of genes was performed with modules having a minimum size of 15 genes, resulting in 67 separate modules using the selected soft thresholding power. Dendrogram plots were also created using the WGCNA package.

#### Gene Ontology and reactome analysis

The Gene Ontology Consortium [32] and PANTHER classification resource [95] were used to identify over-represented terms (biological processes) in each set of predicted cell type enriched genes from the GO ontology (release date 2022–10-13) or reactome (version 77, release date 2021–10-01) databases. Dendrogram plots showing over-represented GO terms in selected cell types were created using the R package clusterProfiler [96, 97].

#### Additional datasets and analysis

Single-cell RNAseq data was downloaded from Tabula Sapiens [7] and analysed using the Seurat package in R [98], which was also used to create the UMAP plots. Information on tissue-enriched gene expression was downloaded from the HPA tissue atlas [6] or GTEx database [27], as collated in the Harminozome database [37].

Stomach cell type classifications from Zhang et al. [11], based on the analysis of gastric mucosae in premalignant and early-malignant lesions, or Busslinger et al. [9] based on the analysis of healthy stomach epithelia were sourced from the respective supplemental material sections (cell type enrichment = log FC gene expression vs. other cell types > 0.58 and > 1.0, respectively [adjusted *p*-value cut off < 0.01], see Additional file 2: Table S1 Key Tab for further details). The statistical significance of overlap between predicted cell type-enriched genes in this study and these scRNAseq studies was calculated using a hypergeometric test (Additional file 4: Fig. S3 E).

The HPA Single Cell Type Section [29] (www.proteinatlas.org/humanproteome/single+cell+type) and Panglao DB [31] were used to identify cell type marker genes (see Additional file 2: Table S1, Key Tab for further details) for comparisons with cell type enriched gene predictions.

#### Visualisation

Unless otherwise indicated, plots and graphs were created using GraphPad Prism version 10, GraphPad Software, Boston, MA, USA, www.graphpad.com. Circular graphs were constructed using the R package *circlize* [99]. The principle component analysis plot was generated using https://biit.cs.ut.ee/clustvis/ [34]. Some figure sections were created with BioRender.com.

### Additional resources

Analysed data for all protein-coding genes is provided on the HPA website: (https://www.proteinatlas.org/humanproteome/tissue+cell+type/stomach). Data for non-coding genes is provided at https://cell-enrichment.shinyapps.io/noncoding_stomach/. The published article includes all datasets generated during this study (Tables S1 and S2). 

## Supplementary Information

**Additional file 1:Supplementary Fig. S1**.

**Additional file 2:Table S1.** Reference transcript selection and analysis criteria. (Tab 1): Correlation coefficient values were calculated between selected Ref.T. to represent constituent stomach cell types. (Tab 2): Correlation coefficient values were calculated between selected Ref.T. and all other sequenced transcripts in GTEx stomach mRNAseq data (Table A) and the mean differential vs. all Ref.T. panels (Table B). Genes classified as enriched in: (Tab 3) parietal cells, (Tab 4) chief cells, (Tab 5) gastric enteroendocrine cells, (Tab 6) gastric mucous cells, (Tab 7) mitotic cells, (Tab 8) endothelial cells, (Tab 9) fibroblasts, (Tab 10) macrophages, (Tab 11) neutrophils, (Tab 12) T-cells and (Tab 13) plasma cells were analysed to identify over-represented terms in the (Table A) gene ontology or (Table **B**). (Tab 14) Comparison of our cell type enrichment predictions with scRNAseq datasets. See key for column details. Related to all Figures.

**Additional file 3: Supplementary Fig. S2**.

**Additional file 4: Supplementary Fig. S3**.

**Additional file 5: Table S2.** Sex stratified subset analysis of cell-enriched genes in human stomach. (Tab 1**)**: Correlation coefficient values were calculated between selected *Ref.T.* to represent constituent stomach cell types in females (Table **A**) or males (Table **B**). (Tab 2) Correlation coefficient values were calculated between selected *Ref.T.* and all other sequenced transcripts in stomach mRNAseq data (GTEx), subdivided into (Table **A**) female or (Table **B**) male only sample sets. See key for column details. *Related to *Fig. 7* and Fig. S4*.

## Data Availability

This study did not generate new unique reagents. • This paper analyses existing, publicly available data from the Genotype-Tissue Expression (GTEx) Project (https://gtexportal.org) with accession number phs000424.v8.p2  [27] and single-cell RNAseq data from Tabula Sapiens  [7] retrieved on 2022/07/29. • All original code has been deposited at GitHub and is publicly available as of the date of publication, link: https://github.com/PhilipDusart/cell-enrichment  [100]. • No additional information should be required to reanalyse the data reported in this paper, but any necessary clarifications or queries can be directed towards the lead contact.

## Abbreviations

CC: Chief cell
Corr: Correlation coefficients
EC: Endothelial cell
FB: Fibroblast
FDR: False discovery rate
GEEC: Gastric enteroendocrine cell
GI: Gastrointestinal
GMC: Gastric mucous cell
GO: Gene Ontology
GTEx: Genotype-Tissue Expression
HPA: Human Protein Atlas
IG: Immunoglobulin
lncRNA: Long non-coding RNA
MC: Macrophage
MTC: Mitotic cell
NP: Neutrophil
PAC: Parietal cell
PC: Plasma cell
Ref.T.: Reference transcripts
scRNAseq: Single-cell RNA sequencing
STD: Standard deviation
TC: T-cell
TPM: Transcripts per million
UMAP: Uniform Manifold Approximation and Projection
WGCNA: Weighted network correlation analysis
