# Supplementary material for: A human stomach cell type transcriptome atlas
Source: BMC Biol. 2024 Feb 14;22:36. doi: 10.1186/s12915-024-01812-5 (PMC10865703; doi:10.1186/s12915-024-01812-5)
Supplement: Supplementary file 1 — Additional file 1:Supplementary Fig. S1. [file 12915_2024_1812_MOESM1_ESM.pdf]

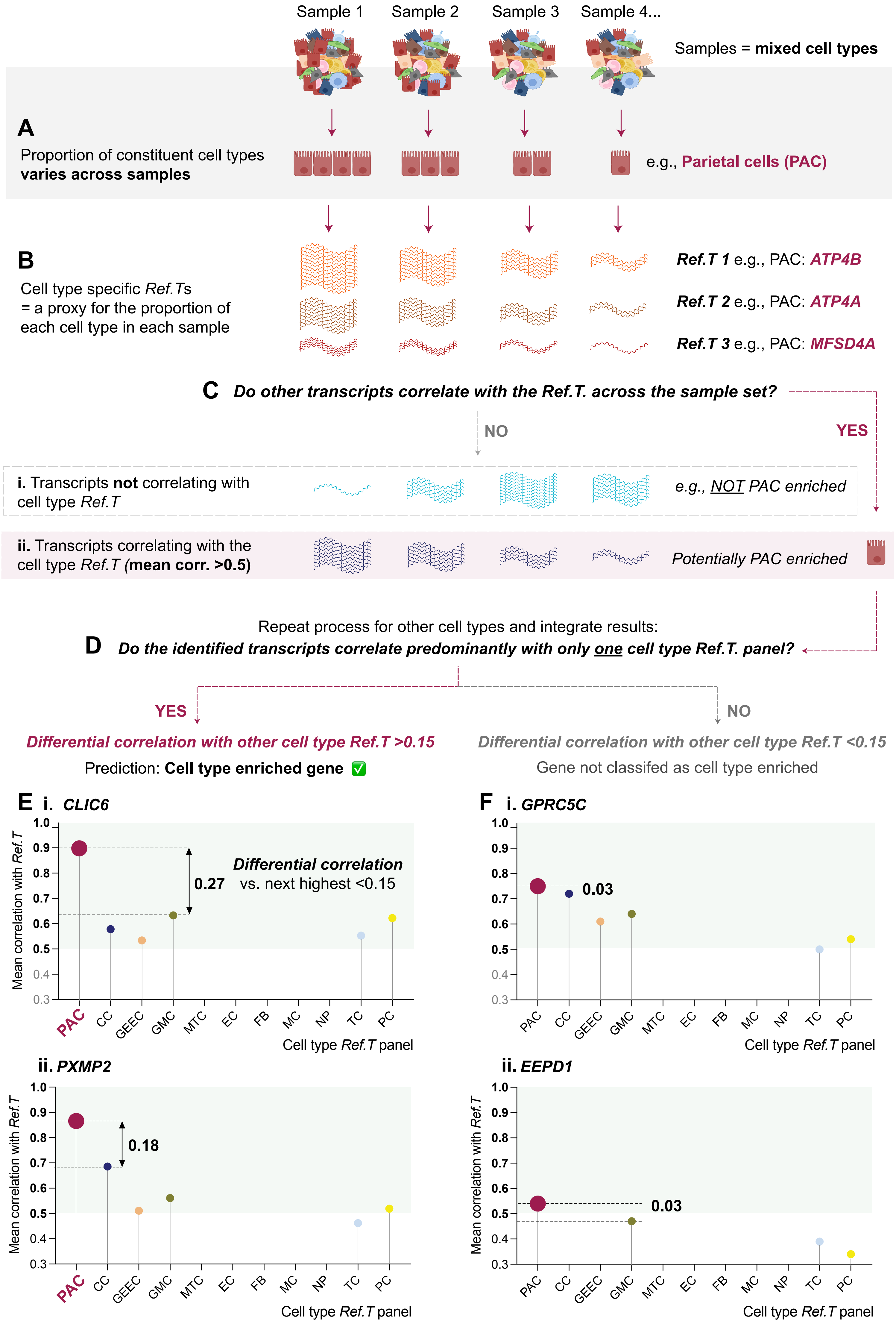

Sample 1
Sample 2
Sample 3
Sample 4...
Samples = mixed cell types
A
Proportion of constituent cell types varies across samples
e.g., Parietal cells (PAC)
B
Cell type specific Ref.Ts = a proxy for the proportion of each cell type in each sample
Ref.T 1 e.g., PAC: ATP4B
Ref.T 2 e.g., PAC: ATP4A
Ref.T 3 e.g., PAC: MFSD4A
C Do other transcripts correlate with the Ref.T. across the sample set?
NO
YES
i. Transcripts not correlating with cell type Ref.T
e.g., NOT PAC enriched
ii. Transcripts correlating with the cell type Ref.T (mean corr. >0.5)
Potentially PAC enriched
Repeat process for other cell types and integrate results:
D Do the identified transcripts correlate predominantly with only one cell type Ref.T. panel?
YES
NO
Differential correlation with other cell type Ref.T >0.15
Prediction: Cell type enriched gene ✅
Differential correlation with other cell type Ref.T <0.15
Gene not classifed as cell type enriched
E i. CLIC6
Mean correlation with Ref.T
Differential correlation
0.27 vs. next highest <0.15
PAC CC GEEC GMC MTC EC FB MC NP TC PC
Cell type Ref.T panel
ii. PXMP2
0.18
F i. GPRC5C
0.03
ii. EEPD1
0.03
1.0 0.9 0.8 0.7 0.6 0.5 0.4 0.3
