# Supplementary figures and images for: A human stomach cell type transcriptome atlas
Source: BMC Biol. 2024 Feb 14;22:36. doi: 10.1186/s12915-024-01812-5 (PMC10865703; doi:10.1186/s12915-024-01812-5)

**A**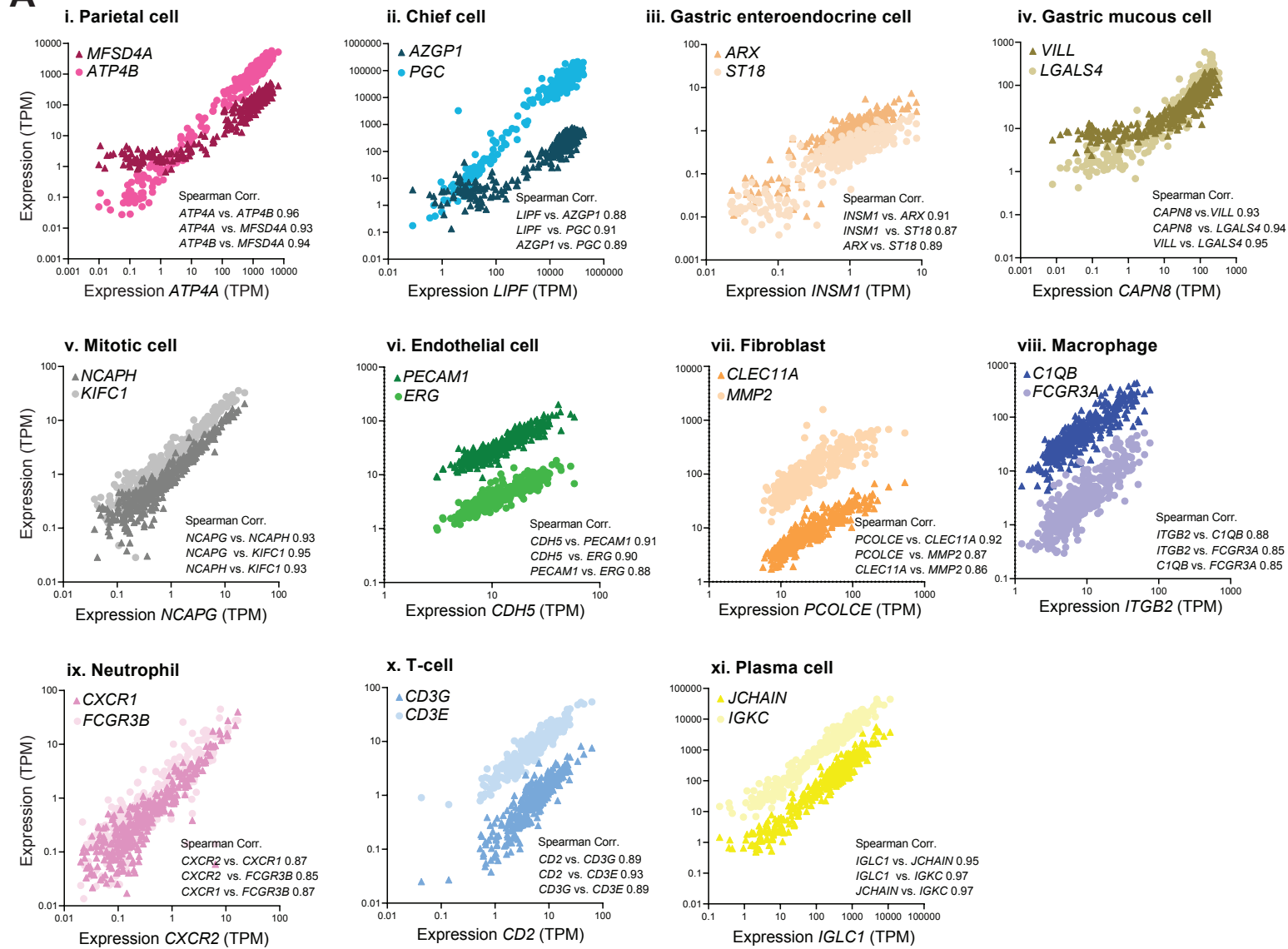**B**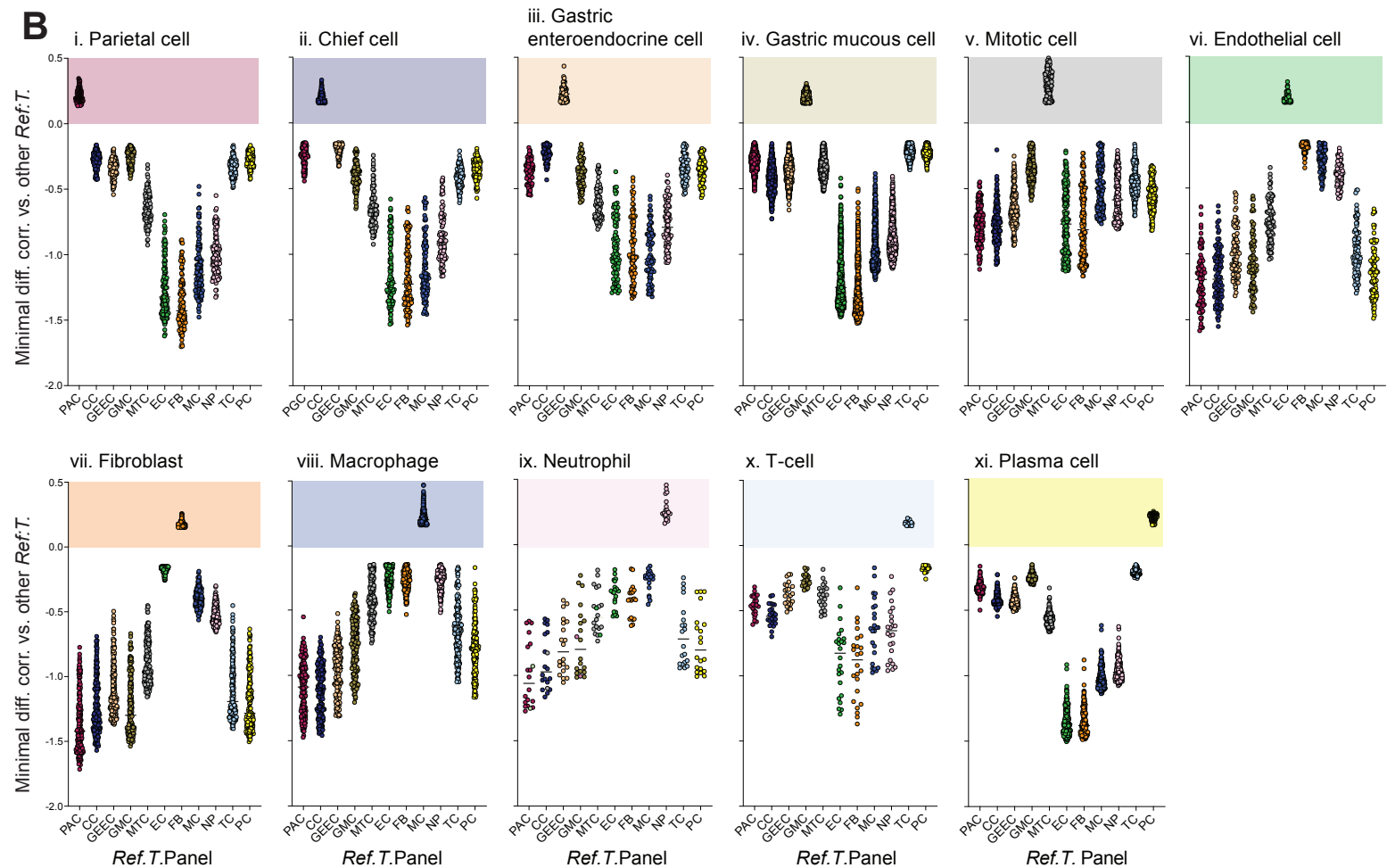

Supplement: Supplementary file 3 — Additional file 3: Supplementary Fig. S2. [file 12915_2024_1812_MOESM3_ESM.pdf]

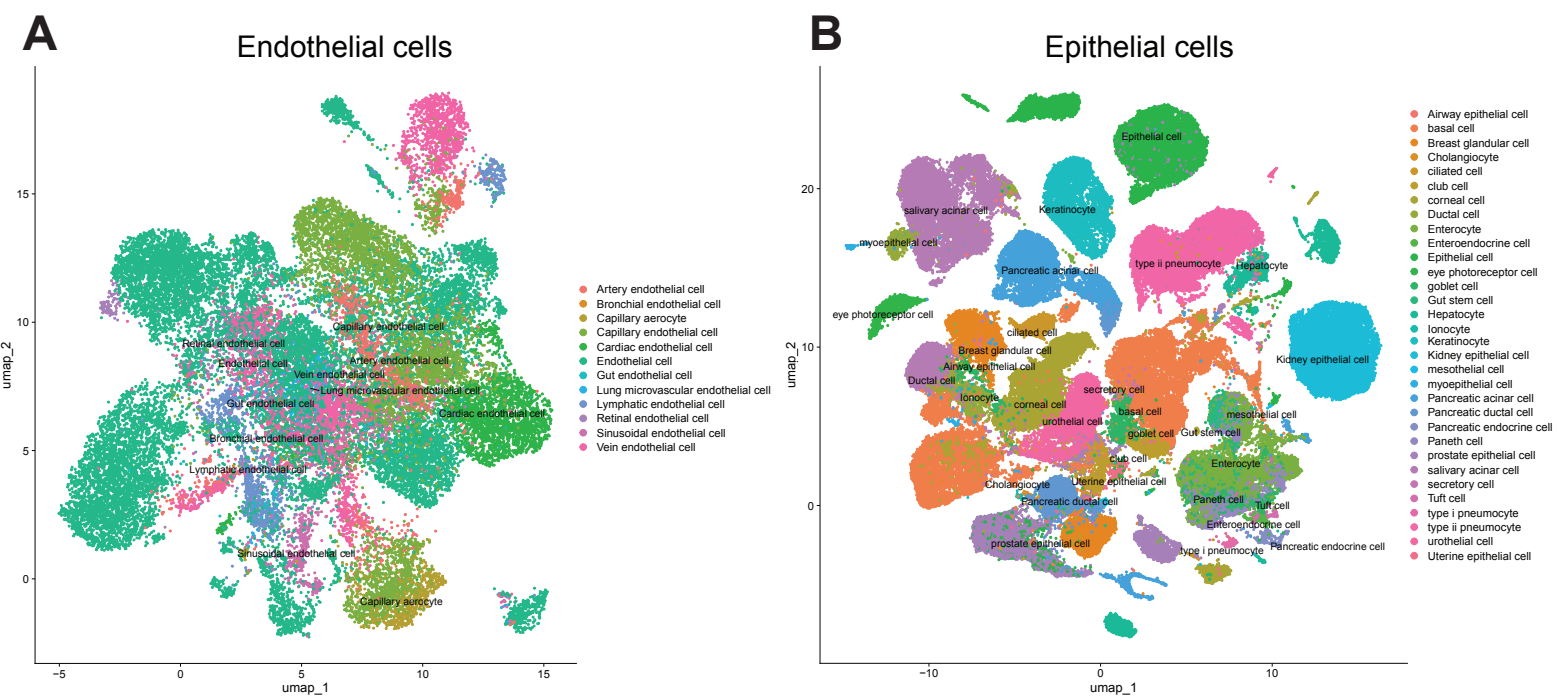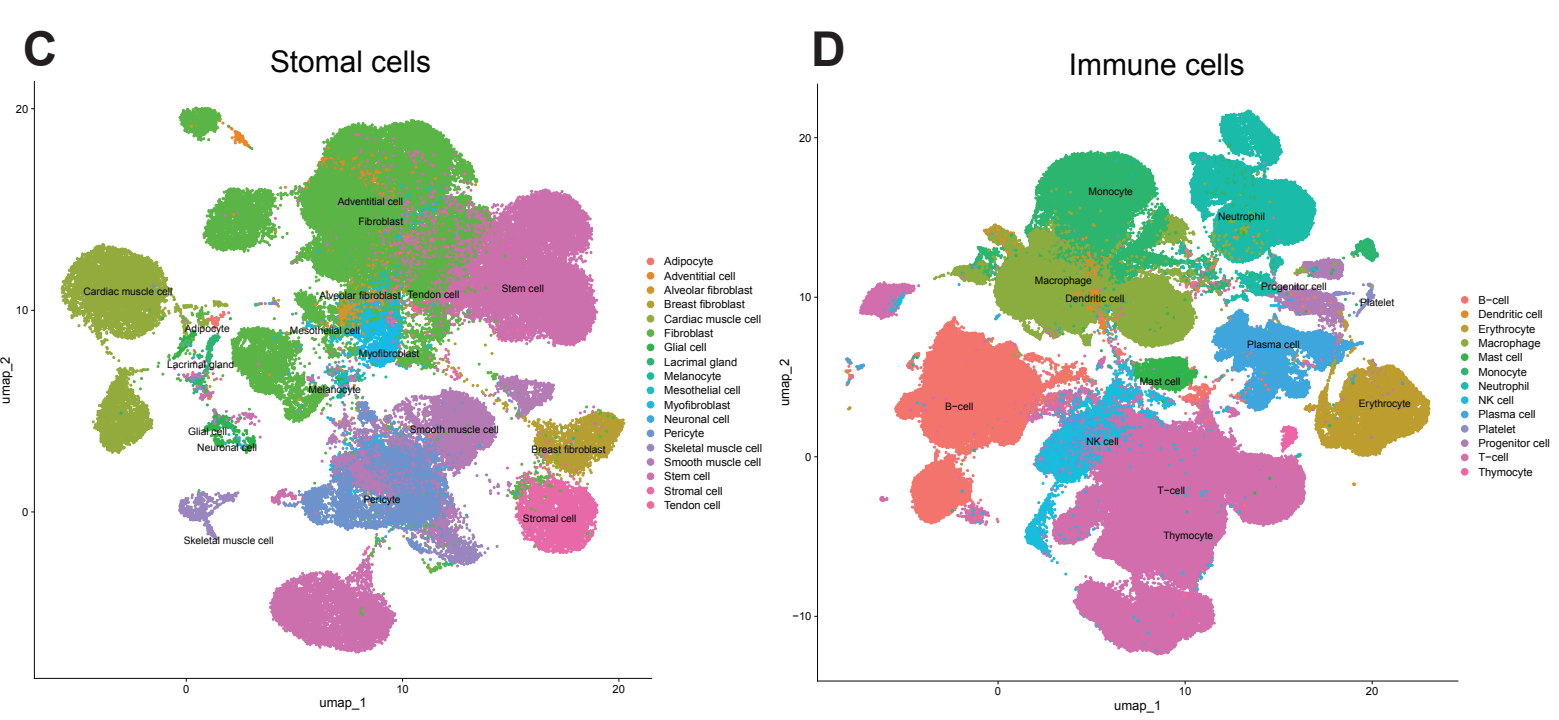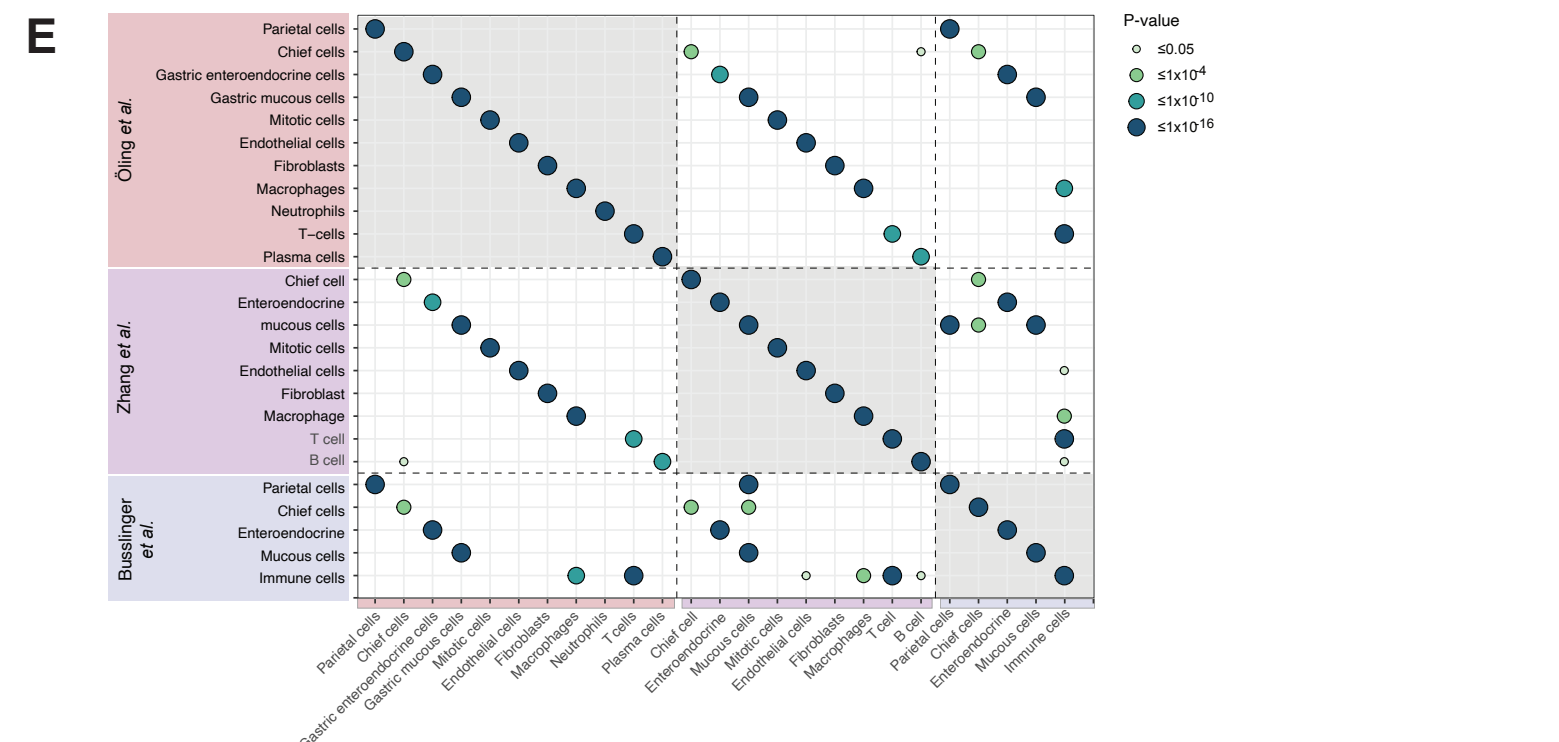

Supplement: Supplementary file 4 — Additional file 4: Supplementary Fig. S3. [file 12915_2024_1812_MOESM4_ESM.pdf]
